# Fibroblast growth factor 10 is a negative regulator of postnatal neurogenesis in the mouse hypothalamus

**DOI:** 10.1242/dev.180950

**Published:** 2020-07-13

**Authors:** Timothy Goodman, Stuart G. Nayar, Shaun Clare, Marta Mikolajczak, Ritva Rice, Suzanne Mansour, Saverio Bellusci, Mohammad K. Hajihosseini

**Affiliations:** 1 School of Biological Sciences, University of East Anglia, Norwich NR4 7TJ, UK; 2 Institute of Biotechnology, University of Helsinki, Viikinkaari 9, Helsinki 00014, Finland; 3 Department of Human Genetics, University of Utah, Salt Lake City, UT 84112-5330, USA; 4 Pediatrics, Saban Research Institute of Children's Hospital Los Angeles, University of California, Los Angeles, CA 90027, USA; 5 Excellence Cluster Cardio Pulmonary System, University Justus Liebig, 35392 Giessen, Germany

**Keywords:** Postnatal neurogenesis, Fibroblast growth factors, Tanycytes, Cell movement, Lineage tracing, Hypothalamus, Mouse

## Abstract

New neurons are generated in the postnatal rodent hypothalamus, with a subset of tanycytes in the third ventricular (3V) wall serving as neural stem/progenitor cells. However, the precise stem cell niche organization, the intermediate steps and the endogenous regulators of postnatal hypothalamic neurogenesis remain elusive. Quantitative lineage-tracing *in vivo* revealed that conditional deletion of fibroblast growth factor 10 (Fgf10) from Fgf10-expressing β-tanycytes at postnatal days (P)4-5 results in the generation of significantly more parenchymal cells by P28, composed mostly of ventromedial and dorsomedial neurons and some glial cells, which persist into adulthood. A closer scrutiny *in vivo* and *ex vivo* revealed that the 3V wall is not static and is amenable to cell movements. Furthermore, normally β-tanycytes give rise to parenchymal cells via an intermediate population of α-tanycytes with transient amplifying cell characteristics. Loss of Fgf10 temporarily attenuates the amplification of β-tanycytes but also appears to delay the exit of their α-tanycyte descendants from the germinal 3V wall. Our findings suggest that transience of cells through the α-tanycyte domain is a key feature, and Fgf10 is a negative regulator of postnatal hypothalamic neurogenesis.

## Abstract (precis)

**Summary:** Generation of new hypothalamic neurons after birth is a multistep process involving cell division and cell movements that are controlled by Fgf10.

## INTRODUCTION

Through a common set of principles, new neurons are continuously generated in the hippocampal dentate gyrus and the ventricular-subventricular zone of the lateral ventricles of the adult rodent brain. In both areas, neural stem cells (NSC) reside in discrete niches and are distinguished from their immediate descendants by quiescence or a slower mitotic rate, as well as differing genetic signatures and immunoprofile. Moreover, adult neurogenesis involves a transient intermediate progenitor cell population and is regulated by a combination of cell-intrinsic and environmental factors, which range from timely expression of fate-determining genes to proximity to blood vessels, and exposure to growth factors and neurotransmitters (Aimone et al., 2014; Bjornsson et al., 2015; Falk and Götz, 2017; Obernier and Alvarez-Buylla, 2019; Ottone et al., 2014; Urbán et al., 2019). Recent evidence shows that a lower level of neurogenesis also occurs postnatally in the hypothalamus (Chaker et al., 2016; Haan et al., 2013; Kokoeva et al., 2005; Lee et al., 2012; Li et al., 2012; Robins et al., 2013a,b). However, the organization of the hypothalamic neurogenic niche, the intermediate steps of neurogenesis and the endogenous factors that regulate it, remain largely undefined.

The mature mouse hypothalamus is approximately contained within rostro-caudal bregma coordinates +0.2 to −2.9 (Franklin and Paxinos, 2007). It is composed of three broad cellular compartments: ependymal cells lining the third ventricle (3V), which include radial-glial like tanycytes; parenchymal neuronal nuclei flanking the 3V with important homoeostatic and adaptive functions; and the median eminence (ME), which harbours a multitude of neurons, glial cells and nerve terminals, with important neuroendocrine and barrier properties (Ebling and Lewis, 2018; Prevot et al., 2018).

A strong body of data shows that hypothalamic neural stem/progenitor cells reside in the ventral part of the 3V ventricular wall, an area enriched in two types of tanycytes – α and β. These are distinguishable by their spatial positioning, radial arbor extension, cilia arrangement and gene marker expression (Goodman and Hajihosseini, 2015; Prevot et al., 2018; Rodriguez et al., 2005). Although postnatal hypothalamic neurogliogenesis can be stimulated by exogenous infusion of growth factors such as BDNF (Pencea et al., 2001), CNTF (Kokoeva et al., 2007), FGF2 (Jourdon et al., 2016; Robins et al., 2013a; Xu et al., 2005) and IGF-1 (Pérez-Martín et al., 2010) into the third or lateral ventricles, its endogenous regulators remain largely unknown.

FGFs function pleiotropically to regulate tissue morphogenesis during embryonic and postnatal development, and to regulate physiological homeostasis in the adult (Brewer et al., 2016; Ornitz and Itoh, 2015). The mammalian FGF signalling apparatus consists of 18 FGF ligands, four membrane-anchored full-length FGF receptors (FGFRs) with a multitude of splice variants, and cell-surface expressed cofactors that determine cell type-specific FGF action (Ornitz and Itoh, 2015). Moreover, there is a growing recognition that some FGFs can function cell-intrinsically (Di Re et al., 2017; Kostas et al., 2018). Previously, we showed that the expression of *Fgf10* in the juvenile and adult hypothalamus is restricted to β-tanycytes and that these cells supply new neurons to the nearby hypothalamic circuits that control energy uptake and expenditure (Haan et al., 2013). However, the role of Fgf10 in β-tanycyte biology or their neurogenic ability remained untested.

Here, we report that conditional deletion of Fgf10 from β-tanycytes *in vivo* enhances postnatal hypothalamic neurogenesis, as evidenced by supernumerary parenchymal neurons. In dissecting the underlying mechanisms, we discovered that normally β-tanycytes give rise to a proliferative transient/intermediate population of α-tanycytes. Loss of Fgf10 diminishes β-tanycyte expansion but also retards the exit of their α-tanycyte descendants from the germinal ependymal layer, thereby possibly creating a greater potential for neural cell production. Collectively, these findings provide novel insights into the niche organization, the intermediate steps and a key endogenous regulator of postnatal hypothalamic neurogenesis. Our results may also help unify the divergent hypotheses regarding the origin and location of stem/intermediate progenitor cells in the postnatal hypothalamus.

## RESULTS

### Deletion of Fgf10 from β-tanycytes amplifies postnatal hypothalamic neurogenesis

**Fig. 1.**
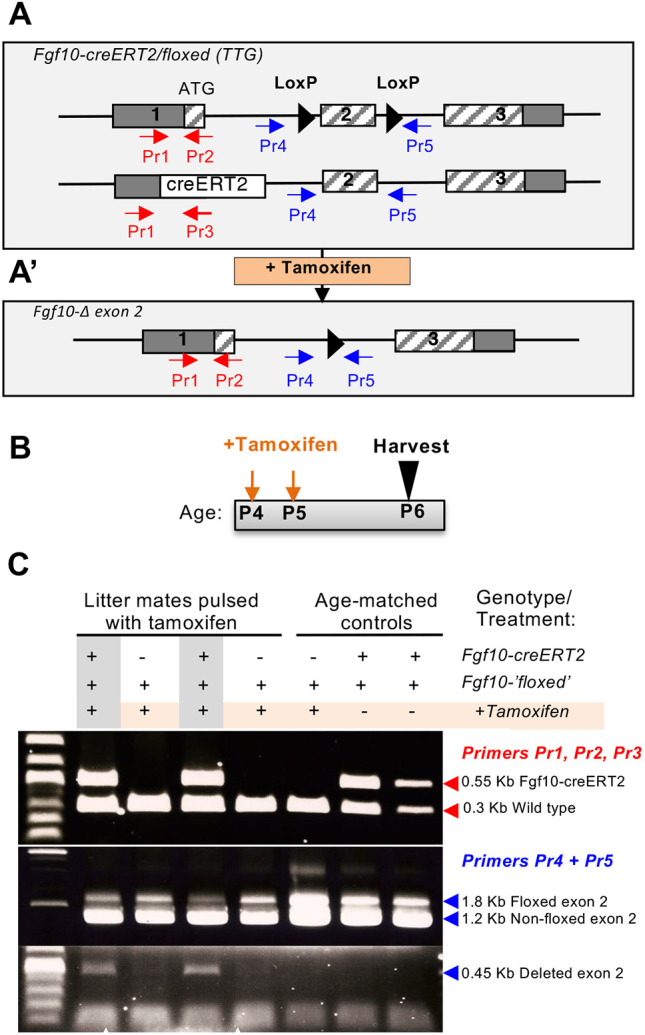
**Conditional deletion of Fgf10 in Fgf10-expressing cells.** (A) Schematic of the Fgf10-creERT2/floxed (TTG) allele, generated through intercrossing of mice carrying the Fgf10-creERT2 (El Agha et al., 2012), Fgf10-floxed (Urness et al., 2010), and R26-flox-STOP-flox-Td-tomato dsRed (not shown) alleles. (A′) Excision of Fgf10 exon 2 upon tamoxifen treatment. (B,C) Experimental paradigm (B) and detection of the alleles (wild-type Fgf10, Fgf10-creERT2, Fgf10-‘floxed’ and Fgf10-exon 2 deletion) by PCR using tissue biopsies from tamoxifen-treated mice and the corresponding primer combinations of primers (Pr) shown in A. Note the absence of the 0.45 Kb exon 2-deleted product in control mice: tamoxifen-treated Fgf10-floxed/+ and non-tamoxifen-treated Fgf10-creERt2/floxed mice (C).

The conserved β-tanycyte-restricted expression of Fgf10 in the murine hypothalamus from early postnatal period [postnatal day (P)8] to adulthood (Fig. S1; Haan et al., 2013; Hajihosseini et al., 2008) led us to hypothesize that Fgf10 plays a crucial role in the neurogenic capacity of β-tanycytes (Haan et al., 2013; Lee et al., 2012). As Fgf10-deficient mice are perinatally lethal and uninformative (Min et al., 1998), we tested this by conditionally deleting Fgf10 from β-tanycytes in young pups and evaluating the fate of their daughter cells. This was achieved by tamoxifen treatment of Fgf10-creERT2/floxed::Rosa26-Tomato-dsRed triple transgenic (TTG) mice, generated through selective breeding. In these mice, a copy of the Fgf10 allele is already abrogated by the CreERT2 knock-in transgene (El Agha et al., 2012) and upon tamoxifen treatment, nuclear translocation of CreERT2 protein excises the ‘floxed’ exon 2 allele (Urness et al., 2010), specifically within Fgf10-expressing cells (Fig. 1A,A′). Simultaneous lineage tracing was afforded by the additional CreERT2-activation of the Tomato-dsRed (Tom) from the Rosa reporter allele. We found that treatment of pups with 100 µg of tamoxifen solution is sufficient to cause rapid deletion of the Fgf10-floxed allele (Fig. 1B,C; Fig. S2), with no deleterious effects. Thus, TTG and control Fgf10-creERT2/+::Rosa26-Tomato-dsRed double transgenic (DTG) litter mates were pulsed at P4 and P5, and the distribution of Tom-expressing (Tom+) cells was quantified within bregma −1.22 to −2.70 in serial brain sections at P6, P12 and P28.

**Fig. 2.**
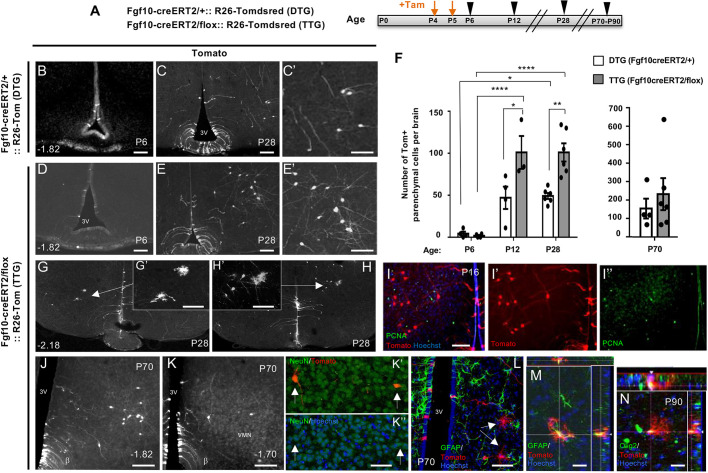
**Overproduction of hypothalamic neural cells upon Fgf10 deletion from β-tanycytes.** (A) Experimental paradigm for simultaneous deletion of Fgf10 and lineage tracing of recombined cells in TTG and DTG mice by tamoxifen treatment at P4/P5 (orange arrows) and subsequent analysis at P6, P12, P28 and P70-P90 (black arrowheads). (B-E′), Representative coronal images of comparable bregma co-ordinates from tamoxifen-treated DTG and TTG brains. (F) Quantifications show a significantly greater abundance of parenchymal Tom+ cells in TTG at P28, with maintenance of parenchymal cells as late as P70. (G-H′), Examples of glial-like Tom+ cells found in TTG brains at P28, (I-I″) Paucity of PCNA expression by lineage-traced parenchymal cells in TTG at P16. (J-N) Persistence of lineage traced cells at P70 (J-L) and P90 (M,N). (J-K″) Examples of neuron-like cells with some verified by NeuN co-expression, as indicated by arrows in K′,K″. (L-N) Examples of glial-like cells (arrows in L), with some expressing GFAP (M) or Olig2 (N) as evident in cut views (insets). DTG: P6 (*n*=4), P12 (*n*=4), P28 (*n*=6), P70 (*n*=4); TTG: P6 (*n*=4), P12 (*n*=3), P28 (*n*=6), P70 (*n*=6), P90 (*n*=1). Data are mean±s.e.m. *P<0.05, **P<0.01, ****P<0.0001 (F, left graph, two-way ANOVA followed by Tukey's test; F, right graph, Student's unpaired two-tailed *t*-test). Numbers at the bottom left of each panel indicate bregma coordinates. Scale bars: 100 µm in B-E′,I-K; 50 µm in G′,H′,K″,L; 25 µm in M,N.

Despite starting with comparable numbers of Tom+ tanycytes (ependymal: 69±12 in DTG versus 43±17 in TTG; i.e. not significantly different; mean±s.e.m.) and negligible Tom+ parenchymal cells in both DTG and TTG at P6, a day after the last tamoxifen dose (parenchymal 4.3±2.4 in DTG versus 1.8±1 in TTG), the conditional deletion of Fgf10 resulted in significantly more parenchymal Tom+ cells by P28, with twice as many cells detectable in TTG compared with DTG (Fig. 2A-F). As in DTG brains, most TTG parenchymal Tom+ cells exhibited clear neuronal morphology, although at P28 only 63±3% of these had differentiated into NeuN+ neurons in TTG, compared with 82±4% in DTG, indicative of a possible delay in cell differentiation. Loss of Fgf10 did not affect the relative contribution of β-tanycytes to appetite/energy balance regulating nuclei of the hypothalamus, as in both genotypes, most lineage-traced Tom+ parenchymal cells were targeted to the ventromedial (VMN) and dorsomedial (DMN) nuclei. Both genotypes contributed a minor number of Tom+ cells to the arcuate nucleus (Fig. S3). However, the significant increase in parenchymal cell number in TTG was evident sooner in VMN (P12) than DMN (P28) (Fig. S3F,G).

In addition to neurons, Tom+ cells with clear glial morphology resembling astrocytes or oligodendrocytes were also evident. These appeared either clustered with immature-looking Tom+ cells or as isolated cell pairs, suggestive of a recent cell division event (Fig. 2G,H; data not shown). However, immunoprobing of P16 brain sections with anti-PCNA antibodies that detect actively cycling cells (Hajihosseini et al., 1996; Kurki et al., 1986) did not reveal many double-labelled (Tom+/PCNA+) cells, glial or otherwise (Fig. 2I).

To test whether lineage-traced cells persist into adulthood, we also examined a cohort of P4/P5 tamoxifen-pulsed mice, aged to P70 (Fig. 2J-N; DTG, *n*=4; TTG, *n*=6) as well as P90 (*n*=1). Parenchymal cells were also numerous in these aged cohorts, even more so than at P28, and more in TTG than DTG (Fig. 2F), although wide variations across the aged animals and the potential for multiple events such as selective survival and/or migration into and out of bregma regions of interest (Haan et al., 2013) during the long gap between P28 and P70 may have masked a significant difference between the two genotypes at this age (Fig. 2F). Nonetheless, even in the aged cohort, some parenchymal cells had clearly differentiated into neurons, oligodendrocytes and astroglial cells marked by NeuN (Rbfox3), Olig2 and GFAP expression, respectively (Fig. 2K-N), indicative of stable integration of the lineage-traced cells.

On face value, detection of significantly more Tom+ parenchymal cells in the hypothalamus of tamoxifen-treated TTG mice by P28 suggests that Fgf10 normally suppresses the rate of neurogenesis during early postnatal life. To understand the causative processes, we first set out to dissect the intermediate steps of postnatal hypothalamic neurogenesis, and then determine how these processes differ in TTG from DTG brains.

### β-Tanycyte-derived α-tanycytes act as intermediate transient cells during postnatal hypothalamic neurogenesis

**Fig. 3.**
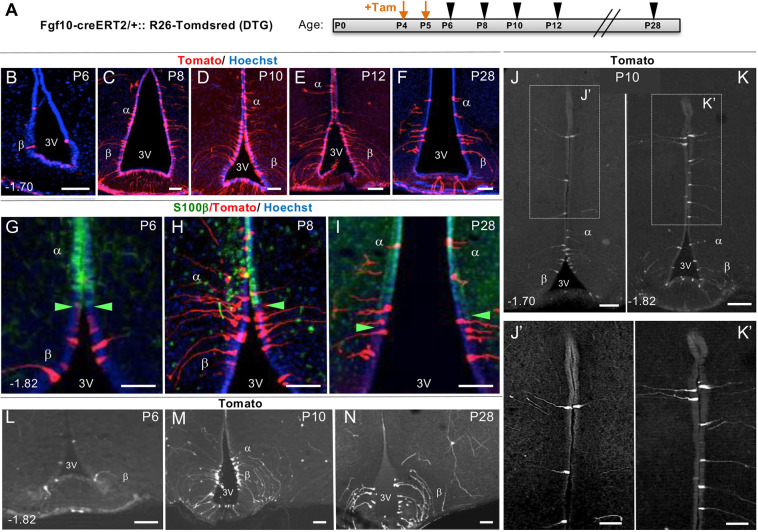
**Trans-compartmental progression of lineage-traced Tomato+ cells from β- to α-tanycyte cell domain.** (A) Experimental paradigm for close scrutiny of Tom+ β-tanycytes and their descendants, labelled at P4/P5 by tamoxifen treatment and traced in cohorts at two-day intervals between P6 and P12, and at P28. (B-F) Low power coronal images showing the overall amplification of Tom+ cells within the ependymal wall. (G-K′) Higher power images showing temporal progression of Tom+ cells across the S100β boundary (green arrowheads; Goodman and Hajihosseini, 2015) (G-I), into and well beyond the classic α-tanycyte domain (J-K′). (L-N) Low power examples of Tom+ parenchymal cells generated by P4/P5 lineage-traced β-tanycytes. Note the scarcity of these cells at P6. Numbers at the bottom left of each panel indicate bregma coordinates. α, α-tanycyte; β, β-tanycyte; 3V, third ventricle. Scale bars: 50 µm in B-I,J′,K′; 100 µm in J,K,L-N.

**Fig. 4.**
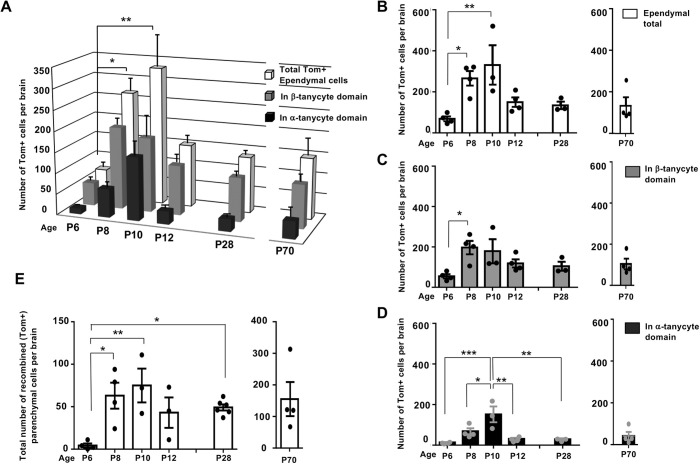
**Differential amplification and retention of lineage-traced β-tanycytes and their progeny, within the ependymal and parenchymal cell compartments.** (A-E) Graphs show the number and spatial distribution of Tom+ cells per hypothalamus in the ependymal (A-D) and parenchymal (E) compartments of DTG pups, pulsed acutely with tamoxifen at P4/P5 and analyzed at the indicated ages. (A) The overall pattern of emergence and decline, split into individual graphs with individual data points for total ependymal cells (B), and cells found in β-tanycyte (C) and α-tanycyte (D) cell domains. Note the lag in the emergence of Tom+ α-tanycytes at P8, their peak at P10, and their subsequent decline in the ependymal compartment by P12 (A,C,D) and maintenance at this level into adulthood (A). (E) Emergence and peak of parenchymal Tom+ cells mirroring that of α-tanycytes between P8 and P10, with a significant retention by P28. Ependyma: all ages *n*=4 except P10, P28 *n*=3. Parenchyma: P6 *n*=4, P8 *n*=4, P10 *n*=3, P12 *n*=3, P28, *n*=6, P70 *n*=4. Data are mean±s.e.m. *P<0.05, **P<0.01, ***P<0.001 (one-way ANOVA followed by Tukey's test).

To delineate the neurogenic steps that intervene β-tanycytes and their parenchymal descendants, we lineage-traced P4/P5 β-tanycytes as described above but scrutinized their progeny at closer time-intervals at P6, P8, P10 and P12, as well as at P28 (Fig. 3A; all ages *n*=4, except P10 and P28 *n*=3). At each stage, we quantified the total number and distribution of Tom+ cells found within the 3V ependyma as well as the flanking parenchyma in serial coronal brain sections (Figs 3 and 4). Furthermore, ependymal Tom+ cells were subclassified as falling either within the β- or α-tanycyte cell domains, using a combination of distinct radial arbor manifested by tomato expression and dorso-ventral positioning within or outside the domain of ependymal S100β expression, the ventral limit of which demarcates the transitional boundary between S100β+/GFAP+/Fgf10− α-tanycyte domain and S100β−/GFAP−/Fgf10+ β-tanycyte domain (Fig. S1; Campbell et al., 2017; Goodman and Hajihosseini, 2015; Hajihosseini et al., 2008; Kaminskas et al., 2019; Mirzadeh et al., 2017).

At P6, a day after the last tamoxifen dose, an average total of 69 (±12) Tom+ tanycytes were found per DTG hypothalamus, predominantly within the β-tanycyte domain. From P8 onwards, amplification of Tom+ β-tanycyte population was accompanied by a gradual appearance of Tom+ cells more dorsally within the domain of S100β expression (Fig. 3B-I). These dorsal cells resembled α-tanycytes, with a short radial process terminating within the hypothalamic parenchyma (Fig. 3H,I), and a subset expressed S100β (see below). Remarkably, at P10 a few Tom+ α-tanycyte-resembling cells were found well beyond the classic α-tanycyte domain (Fig. 3J-K′) (Rodriguez et al., 2005), supporting the notion that during early postnatal life, walls of the 3V are amenable to cell movement/cell mixing.

Detailed quantifications revealed a lag between the peak of lineage-traced Tom+ β-tanycytes at P8 (197±33) versus α-tanycytes at P10 (152±39) (Fig. 4A-D). Thereafter, both populations declined, with 58% retention for β-tanycytes, but only 19% retention for α-tanycytes by P28 (Fig. 4A; C versus D), with some of the retained α-tanycytes upregulating S100β (Fig. S4). The ectopically located ‘α-tanycytes’ (Fig. 3J,K) had disappeared altogether from the ependymal layer. We established that the disappearance of α-tanycytes, ectopic or otherwise, is not due to cell death. Anti-cleaved caspase3 immunolabelling of either wild-type or DTG sections at P10, corresponding to decline of Tom+ α-tanycytes (Fig. 4A,D), revealed negligible cell death in the ependymal layer (Fig. S5), in agreement with previous cell death measurements in the postnatal hypothalamus (Guyenet et al., 2013).

In accordance with the expansion of Tom+ α-tanycytes at P8, a significant number of Tom+ parenchymal cells also emerged from P8 onwards in the adjacent VMN and DMN nuclei, with a subset differentiating into NeuN+ neurons (Fig. 3L-N; Fig. 4E; Fig. S3A).

### Differential cell proliferation and cell dispersion dynamics within β- and α-tanycyte domains

**Fig. 5.**
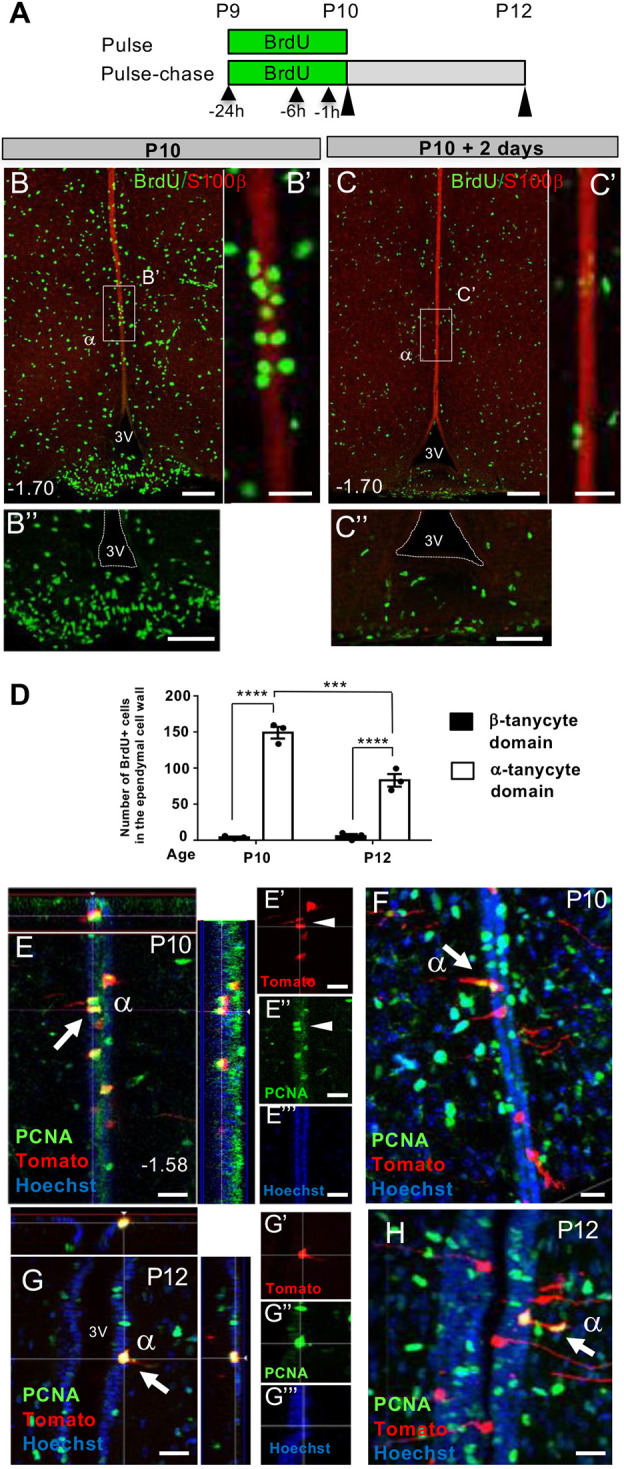
**Dynamics and appearance of proliferating cells in β- and α- tanycyte cell domains.** (A) Experimental paradigm consisting of three BrdU pulses between P9-P10 (black arrowheads) followed by acute (P10) or 2-day chase (P12) (black elongated arrowheads) to assess selective distribution and retention of BrdU-incorporating ependymal cells within β- versus α-tanycyte domains. (B,C″) Coronal images from comparable bregma levels showing a cluster of BrdU+ cells within the α-tanycyte S100β^+^ cell domain (B,B′), and their disappearance by P12 (C,C′). B″ and C″ show high power images of B and C, showing scarcity of BrdU+ ependymal cells in the β-tanycyte S100β^−^ cell compartment. (D) Quantification of BrdU+ cells in the respective cell domains contained between bregma −1.34 and −2.54. (E-H) Examples of PCNA+/Tom+ α-tanycyte cell pairs at P10 and P12. Cells in E-G (arrow and arrowheads) show symmetric divisions, generating two ependymal daughters, whereas cells in F and H (arrowed) appear to generate at least one parenchymal daughter, suggestive of asymmetric cell division/fate. *n*=3 at each time point. Data are mean±s.e.m. ***P<0.001, ****P<0.0001 (two-way ANOVA followed by Tukey's test). α, α-tanycyte; 3V, third ventricle. Numbers at the bottom left of each panel indicate bregma coordinates. Scale bars: 100 µm in B,B″,C,C″; 25 µm in B′,C′,E-H.

We obtained more evidence for transience of cells from the α-tanycyte domain whilst comparing the distribution of 5-bromo-2′-deoxyuridine (BrdU)-incorporating ependymal cells within α-tanycyte (S100β-positive) versus β-tanycyte (S100β-negative) domains. For this, wild-type mice were analyzed, as the harsh HCl-pretreatment required for anti-BrdU immunolabelling appeared to interfere with immunodetection of Tomato-ds red in tamoxifen-treated BrdU-pulsed brain DTG sections. Thus, DTG mice were BrdU-pulsed three times intraperitoneally at P9 (*n*=6) and chased at P10 (*n*=3) and P12 (*n*=3) (Fig. 5A) to capture the peak and subsequent decline of Tom+ α-tanycytes described in Fig. 4A and D. Remarkably, a tight cluster of BrdU+ cells was evident within the S-100β+ α-tanycyte domain at P10, and these cells were depleted by P12 (Fig. 5B,B′ versus C,C′). Furthermore, compared with a high number of BrdU+ cell numbers in the α-tanycyte domain (149±8), significantly fewer β-tanycytes incorporated BrdU within this narrow pulse window (Fig. 5B″ versus C″,D), which is reminiscent of slow dividing stem cells as opposed to proliferative lineage amplifying cells in other neurogenic niches (Codega et al., 2014).

To visualize the proliferation of Tom+ ependymal cells directly, we immunostained sections of tamoxifen-treated P4/P5 DTG brain for PCNA at P10 and P12 and found clear co-localization of PCNA with Tom-dsRed in a subset of Tom+ α-tanycytes (Fig. 5E-H). Interestingly, these appeared as cell pairs contained either entirely within the ependymal layer (Fig. 5E,G), or with one or both daughter cells appearing to exit it (Fig. 5F,H), reminiscent of symmetric and asymmetric cell divisions/cell fate, respectively.

**Fig. 6.**
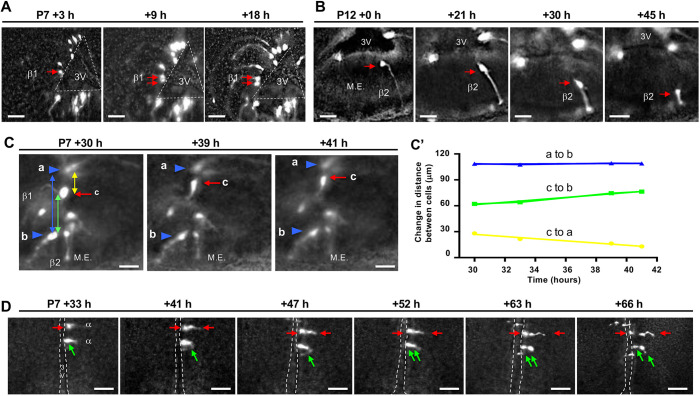
**Division and translocation of β- and α-tanycytes *ex vivo.*** (A-D) Representative time-lapse images of lineage-traced Tom+ β-tanycytes (A-C) and α-tanycytes (D) in organotypic slice cultures. All images are derived from *in vivo* tamoxifen-pulsed P4/P5 brain, analyzed *ex vivo* starting at P7 or P12 for up to 96 h. (A) Cell division (red arrows) adjacent to 3V wall (dashed lines) in β1-tanycyte domain. (B) Delamination of an apical process from the ependymal wall and/or translocation of cell body towards the pial surface within β2-tanycytes domain (red arrow), resembling intra-kinetic nuclear migrations of embryonic ventricular zone cells. (C,C′) Ventro-dorsal translocation of a lineage-traced cell (cell ‘c’, red arrow) within the ventricular wall (C) and quantification of the distance travelled by cell ‘c’ away from cell ‘b’ (green line) towards cell ‘a’ (yellow line) (C′). Cell movement is not because of tissue shrinkage as the distance between cells ‘a’ and ‘b’ (blue line, ∼110 µm) remains relatively unchanged. Blue arrowheads indicate the position of cells ‘a’ and ‘b’. (D) Two independent cell division events by Tom+ α-tanycytes (red and green arrows). Note the asymmetry and subsequent lateral migration of daughters of these divisions into the neighbouring parenchyma. α, α-tanycyte; β1, β1-tanycyte; β2, β2-tanycyte; 3V, third ventricle; M.E., median eminence. See also Movie 1. Scale bars: 20 µm.

In a complementary set of experiments, we were able to visualize clear motility and division of ependymal cells in organotypic brain slice cultures derived from pups that were tamoxifen-pulsed at P4/P5 *in vivo* and analyzed either at P7 or P12 over a 96 h period, *ex vivo* (Fig. 6A,C,D; Movie 1). These analyses revealed division of Tom+ cells within the β- and α-tanycyte domains, as well as dorsal movement of β1-tanycytes and a peculiar delamination of β2-tanycytes from the ventricular surface of the ME (Fig. 6B).

We also established that the trans-compartmental cell movements described above are not peculiar to the young (P4/P5) hypothalamus, as similar dynamics of Tom+ β- and α-tanycyte emergence accompanied by the emergence of parenchymal cells were also observed in P44/P46 tamoxifen-treated mice, analyzed either acutely or after a 9-day delay at P55 (Fig. S6A-G). Moreover, in these older lineage-tracing experiments, parenchymal Tom+ cells were targeted to DMN, VMN and the arcuate nucleus (Fig. S6E-G).

Taken together, and consistent with differential expression of neural stem/progenitor markers in β- and α-tanycyte cell domains (Haan et al., 2013; Fig. S1), these findings strongly suggest that Fgf10+ β-tanycytes act as a putative stem cell population in the postnatal hypothalamus and, remarkably, give rise to parenchymal cells via descendants that transit through the α-tanycyte domain. However, we cannot exclude some direct contribution from β-tanycytes to parenchymal cells or that a small subset of parenchymal Tom+ cells arose directly from endogenous Fgf10/creERT2 expression, i.e. scant cells that were not detected by *in situ* hybridization for Fgf10 (Hajihosseini et al., 2008).

### Loss of Fgf10 temporarily retards β-tanycyte amplification but also delays the transience of α-tanycytes

**Fig. 7.**
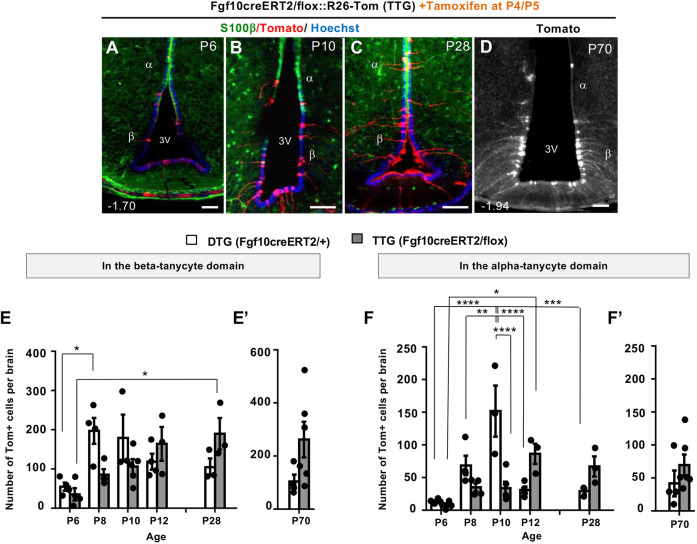
**Loss of Fgf10 differentially impacts tanycyte subtypes.** (A-D) Temporal emergence of Tom+ tanycytes in tamoxifen-treated TTG brains from P6 to P28 (A-C), and their maintenance at P70 (D), mirroring that of DTG (Fig. 2B,C). (E,F′), Comparative quantifications between DTG and TTG reveals an initial general lag in the expansion of β-tanycytes in TTG, followed by a significant delay in the emergence of α-tanycytes by P10. Note, subsequent to P10, α-tanycytes show significant persistence in the ependyma of TTG compared with DTG. DTG all ages *n*=4 except P10, P28 *n*=3; TTG P6 *n*=4, P8 *n*=4, P10 *n*=5, P12 *n*=3, P28 *n*=3 and P70 *n*=6. Data are mean±s.e.m. *P<0.05, **P<0.01, ***P<0.001, ****P<0.0001 (E,F, two-way ANOVA followed by Tukey's test; E′,F′, Student's unpaired two-tailed *t*-test). α, α-tanycyte; β, β-tanycyte; 3V, third ventricle. Numbers at the bottom left of each panel indicate bregma coordinates. Scale bars: 50 µm.

A comparison of DTG and TTG hypothalami at 2-day intervals between P6 and P8 (Fig. 7A-F), showed that the normal amplification of Tom+ β-tanycytes observed in DTG is somewhat retarded in TTG brains (Fig. 7E). This was also reflected in a relative scarcity of TTG Tom+ α-tanycytes in the ependymal layer at P10 (Fig. 7F). By P12, however, although Tom+ β-tanycyte numbers had equalized across the two genotypes and showed no significant difference thereafter, significantly more Tom+ α-tanycytes were present in the S100β+ ependymal cell compartment in TTG. By P28, 2.3 times as many Tom+ α-tanycytes were retained in TTG, compared with DTG (Fig. 7F). To check whether the greater α-tanycyte abundance in TTG is due to cell death in DTG, we evaluated cell death using anti-cleaved caspase3 immunolabelling, only to discover a comparable low number of dying cells across the two genotypes (data not shown).

In the aged cohort (P70; Fig. 7D,E′,F′), β-tanycytes appeared to be more numerous in TTG than DTG, but α-tanycyte numbers were similar across the two genotypes. However, as with parenchymal cell number comparisons (Fig. 2F), our current poor understanding of the dynamics of hypothalamic neurogenesis progression with age, and the long gap between P28 and P70, precludes a meaningful biological comparison of cell population dynamics across the two ages.

## DISCUSSION

**Fig. 8.**
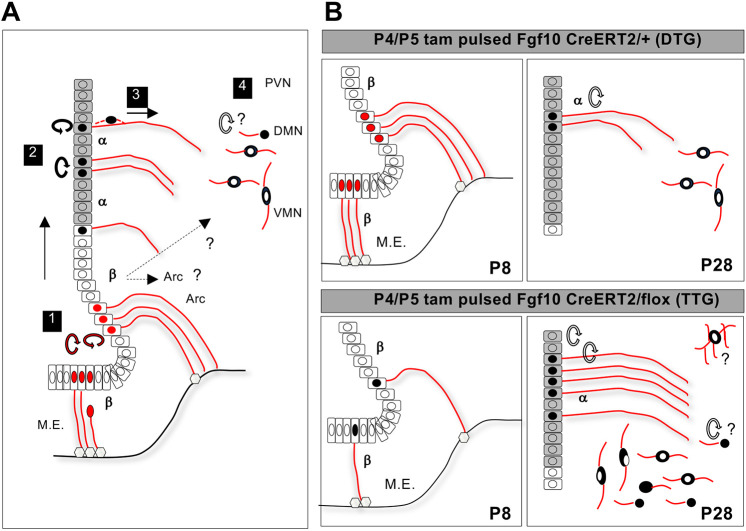
**Proposed model for intermediate steps of early postnatal hypothalamic neurogenesis, and the regulatory role/s of Fgf10.** (A) Intermediate steps. Step 1, β-tanycytes (β) with radial processes terminating onto the outer capillary plexus of the ME (grey hexagons) or the pial surface can delaminate (Fig. 6B) and divide either symmetrically to self-renew (Hajihosseini et al., 2008) or asymmetrically to generate cells that migrate dorsally within the 3V ependymal wall (Fig. 6A) into the zone of S100β expression (grey cells), which coincides with the α-tanycyte (α) cell domain. Step 2, the newly-generated α-tanycytes amplify in number in the germinal ependymal layer, through both symmetric and asymmetrical cell divisions. Step 3, asymmetric divisions generate daughters that migrate into the neighbouring parenchyma (Movie 1). Step 4, the descendants of α-tanycytes differentiate mostly into neurons but a subset may amplify further within the parenchymal compartment (indicated by a question mark). Dashed arrows indicate the possibility that some neurons may be derived directly from β-tanycytes, particularly in the arcuate nucleus (Arc). Cells with red nuclei denote Fgf10-expressing cells; cells with black nuclei denote cells that have turned off Fgf10 expression (A, α-tanycytes) or lack Fgf10 expression (B, lower panel) following its conditional deletion in TTG mice. (B) Regulatory function/s of Fgf10, the expression of which is normally limited to β-tanycytes, in which it may maintain an undifferentiated state and/or suppress cell proliferation. Despite the temporary retardation in Fgf10-deficient β-tanycytes (lower panel), more α-tanycytes eventually result, either because the residual β-tanycytes are pushed into differentiation or escape from an inhibition on cell proliferation. However, loss of Fgf10 may also impact cell movement and cell fate and trap more α-tanycytes within the germinal layer, which, as a putative transient amplifying population, go on to generate neurons and glial cells or progenitors that amplify further in the absence of Fgf10 (question marks). Diagrams are not to scale. M.E., median eminence.

Previously, Fgf10-expressing β-tanycytes were shown to supply the postnatal mouse hypothalamus with new neurons (Haan et al., 2013). However, the putative role/s of Fgf10 in this process, as well as the underlying mechanisms, were unknown. Here, we report that Fgf10 normally represses postnatal hypothalamic neurogenesis, as more parenchymal cells are found upon its deletion from β-tanycytes. We discovered that Fgf10+ β-tanycytes normally give rise to a proliferative population of α-tanycytes, with characteristics of transient amplifying/intermediate progenitor cells. α-Tanycytes then appear to undergo symmetric as well as asymmetric divisions to generate new parenchymal daughter cells. Loss of Fgf10 retards β-tanycyte amplification, but this is compensated later by a greater retention of β-tanycytes and their transient amplifying progeny within the 3V ependymal cell layer, suggesting that Fgf10 may also regulate the movement of tanycytes. Persistence of Fgf10-deficient transient amplifying α-tanycytes in the germinal ependymal compartment could also contribute to a lineage amplification and explain the eventual discovery of more parenchymal cells in the neighbouring VMN and DMN (Fig. 8).

### Walls of the 3V – compartmentalized but dynamic

Active and directional flow of the cerebrospinal fluid in the ventral part of the 3V is well documented (Faubel et al., 2016), but the 3V cell wall itself has often been regarded as a mere physical barrier separating the hypothalamic parenchyma and the ME from the ventricular cavity. Moreover, numerous studies have shown that the ependymal cells in the 3V wall fall into distinct compartments with respect to gene expression, morphology and function, conjuring the notion that they too are rather static in fate and function. For example, cells bearing primary cilia are strictly restricted to the S100β−/GFAP− domain in the floor of the 3V, whilst cells with motile cilia are prevalent dorsally in the S100β+/GFAP+ domain, corresponding to α-tanycytes and ependymocytes (Mirzadeh et al., 2017). In the present study, we evidenced cell division and at least three types of ependymal cell movements: a ventro-dorsal cell translocation from the S100β− β-tanycyte domain into more dorsal S100β+ cell territory; migration of α-tanycyte daughter cells into the hypothalamic parenchyma along the radial processes of their parental cell; and delamination of some β2-tanycytes from the ventricular surface within the ME, resembling basal radial glia in the embryonic cerebral cortex (Vaid et al., 2018). Cell movements along other axes – rostro-caudal or indeed in the dorso-ventral direction – may also be possible, with the latter evidenced by Robins et al. (2013a).

Fluidity of germinal epithelia has long been recognized, but the concept has hitherto not been extended to the 3V wall. Examples include the stochastic cell mixing and dispersion of progenitor cells within the ventricular zone of the developing rodent cerebral cortex (Walsh and Cepko, 1993) and the displacement of newly-generated transient amplifying cells away from the stem cell niche located at the base of the gut epithelial crypt (Ritsma et al., 2014) – not too dissimilar in architecture from the floor of the 3V. Our findings, therefore, challenge a static view of the 3V wall and show that a subset of its cells can translocate across marker boundaries and even change fate, at least as late as the 6th postnatal week (Fig. S6). Further studies are required to delineate the fundamentals and regulators of this fluidity, i.e. whether division and passive cell displacement alone is a sufficient explanation, or whether the fluidity involves discrete conduits, instructive signals or physiological demands on the mediobasal hypothalamus.

### Importance of postnatally-generated neuronal and glial cohorts

Our previous studies focused on the cumulative contribution of Fgf10-expressing β-tanycytes to the arcuate nucleus in constitutive Fgf10-*lacZ* reporter mice (Haan et al., 2013). However, the present study complements the findings of other researchers (Chaker et al., 2016) to show that the VMN, DMN and paraventricular nucleus (PVN) (not measured by us) are the predominant population of postnatally-generated hypothalamic neurons. In the present study, we also noted a few lineage-traced cells in the lateral nucleus (data not shown). Deletion of Fgf10, although significantly increasing parenchymal cell number, did not alter this predominance. In line with our proposed model of neurogenesis (Fig. 8), one explanation may be the ventro-dorsal/medio-lateral anatomical register of the transient amplifying α-tanycyte cell population and the ectopic ‘α-tanycytes’ (Fig. 3J-K) with these nuclei. Previous postulations (Bolborea and Dale, 2013) and our observation that daughters of α-tanycytes use the radial arms of their parents to migrate laterally into the hypothalamic parenchyma (Movie 1) supports this argument. Parenchymal descendants of α-tanycytes need not be localized to the immediate vicinity of the 3V wall though (Movie 1), as we found examples of tanycyte processes extending for hundreds of micrometres into the hypothalamic parenchyma (Fig. S7B). However, we found no evidence for transfer of tomato-dsRed from end-terminals of α- or β-tanycytes to parenchymal cells as an explanation for the occurrence of Tom+ parenchymal cells (Fig. S7).

An alternative explanation is that lineage-traced Tom+ α-tanycytes fell within regionalized sub-compartments of the 3V wall (Campbell et al., 2017) that specify neuronal type/fate, akin to compartmentalized neurogenesis in the ventricular-subventricular zone of the lateral ventricles (Merkle et al., 2007).

Nestin-creER lineage tracing of β-tanycytes at P4 or P7 (Lee et al., 2012) showed a contribution to neurons of ME (Lee et al., 2012). We did not see such a contribution by Fgf10+ β-tanycytes lineage-traced at P4/P5 or P44/P45, although this difference may be explained by the reported heterogeneity of this cell population, both with respect to nestin and Fgf10 expression (Haan et al., 2013). Similarly, tanycytes were shown to be incapable of replenishing the oligodendrocyte progenitor pool after experimental depletion of NG2+ cells from the mediobasal hypothalamus (Robins et al., 2013c). However, our studies here show that they can indeed generate astrocytes and Olig2+ oligodendrocytes under normal conditions.

We did not phenotype neurotransmitter expression or connectivity of the supernumerary hypothalamic neurons resulting from conditional Fgf10 deletion, and the transduced mice themselves did not manifest any discernible defects, although we had deliberately aimed for acute tamoxifen induction to lineage trace a small set of Fgf10+ β-tanycytes and these could have been replenished in time by non-transduced cells. Nonetheless, the level of neuronal overproduction, even under this acute paradigm, and the retention of P4/P5 lineage-traced cells into adulthood (P70) is impressive. Moreover, considering that VMN and DMN harbour important second-order neurons in the networks that regulate appetite and/or energy expenditure (Schneeberger et al., 2014), the significance of supernumerary neurons in these nuclei may only become apparent after prolonged ageing, sustained Fgf10 deletion, and/or physiological and metabolic stress.

### Implications for cell identity and organization of the hypothalamic neurogenic niche

The exact location of stem/progenitor cells in the postnatal hypothalamus has been the subject of much debate, i.e. whether β- or α-tanycytes are the true stem cells and/or whether secondary stem cell populations exists within the hypothalamic parenchyma proper (Li et al., 2012; Robins et al., 2013b). Our proposed model (Fig. 8) partially unifies these views and places β-tanycytes at the top of the neurogenic lineage, with α-tanycytes acting as their transient amplifying descendants, and the potential for secondary amplification of the lineage by progenitors supplied to the hypothalamic parenchymal compartment being a strong possibility.

Lineage tracing using other β- or α-tanycyte-specific reporter models may reveal further heterogeneity. Evidence to place Fgf10+ β-tanycytes in this order of hierarchy includes a slower rate of BrdU incorporation *in vivo*, in acute pulsing paradigms when compared to α-tanycytes; the ability to generate fast-dividing α-tanycyte descendants; and the ability to directly or indirectly generate all three neural cell lineages *in vivo*. The expression of Fgf10 itself may add to this accolade, as recent studies in the chick imply that embryonic Fgf10+ cells of the hypothalamus may act as founders for its postnatal stem cells (Fu et al., 2017).

### Fgf10 as a cell-intrinsic negative regulator of neurogenesis

Pioneering studies have shown that exogenous stimulation of 3V wall cells with BDNF, CNTF, IGF-1 and FGF2 can stimulate cell proliferation and neurogliogenesis, with some resulting in profound effects on body weight (Kokoeva et al., 2005). The endogenous role of these signalling pathways is beginning to be explored. For example, Chaker et al. showed that conditional deletion of IGF-1 receptor (IGF-1R) from a wide cohort of nestin-expressing tanycytes enhances hypothalamic cell production as late as P80 (Chaker et al., 2016). Postnatal hypothalamic neurogenesis is also amenable to dietary interventions (Lee et al., 2012) and photo-period-dependent signals (Batailler et al., 2018, 2016).

Here, we explored the role of Fgf10, which in the postnatal hypothalamus is overwhelmingly restricted to β-tanycytes (Hajihosseini et al., 2008). The normal role of Fgf10 in these cells could be the maintenance of an undifferentiated state alone, or coupled to an active role in suppression of cell proliferation, such that deletion of Fgf10 would then prompt greater β-tanycyte differentiation into its lineage derivatives, or remove a brake on cell proliferation, with the net result of lineage amplification. Nonetheless, the finding that Fgf10 is a negative regulator of neurogenesis is rather surprising, as it is typically associated with positive cell growth in many non-neural embryonic tissues (Min et al., 1998). Negative regulation was noted in embryonic cerebral cortex, where Fgf10 appears to be expressed during a narrow time-window to regulate the transition of neuroepithelial cells into radial glia; loss of Fgf10 initially delays cortical neurogenesis but eventually results in an overproduction of cortical neurons (Sahara and O'Leary, 2009). Hypothalamic Fgf10 expression, however, persists throughout adult life, suggestive of an ongoing role (Fig. S1A; Hajihosseini et al., 2008).

The puzzling dichotomy of Fgf10 function in hypothalamus versus non-neural tissues, and its opposite role to that of FGF2 in postnatal hypothalamus (Jourdon et al., 2016; Robins et al., 2013a; Xu et al., 2005), may be explained by at least two factors. First, FGF2 and FGF10 engage different sets of the alternatively-spliced FGF receptors – the so called IIIc and IIIb isoforms, respectively (Zhang et al., 2006). The receptors for FGF2 – FgfR1-IIIc and FgfR2-IIIc isoforms – are expressed by β-tanycytes (Kaminskas et al., 2019), but the cognate receptor for FGF10 – FgfR2-IIIb – is absent from the hypothalamus altogether (Hajihosseini et al., 2008). Second, the recent discovery that FGF10 harbours nuclear localization motifs that facilitate its nuclear/nucleolar targeting has opened up the possibility that FGF10 can also function non-canonically to drive cell-autonomous gene-regulatory effects within Fgf10+ cells themselves (Mikolajczak et al., 2016). Therefore, in the absence of its receptor from the hypothalamus, a cell-intrinsic receptor-independent role for Fgf10 in β-tanycytes is plausible. Future loss- and gain-of-function studies *in vivo* focusing on this mode of FGF10 function may reveal more about its molecular significance. Similarly, use of dual reporter systems such as Rosa-mT/mG mice may prove more effective in dissecting the lineage relationships that we described here.

In summary, we show that postnatal hypothalamic neurogenesis is a multistep process involving discrete cell movements and generation of an intermediate/transient progenitor cell population. Moreover, we identify Fgf10 as a cell-intrinsic suppressor of neurogenesis.

## MATERIALS AND METHODS

### Animal models

Fgf10-CreERT2/+::Rosa26-loxP-STOP-LoxP-Tomato-dsRed double transgenic (DTG) mice were genotyped and used to lineage trace Fgf10-expressing tanycytes, as previously described (Haan et al., 2013). Fgf10-deficient tanycytes were generated and lineage traced by tamoxifen treatment of Fgf10-CreERT2/flox::Rosa26 loxP-STOP-LoxP-Tomato-dsRed triple transgenic (TTG) mice (see below). In these mice, one copy of Fgf10 exon 1 is already abrogated through the knock-in creERT2 transgene (El Agha et al., 2012) but with no obvious phenotype, whilst tamoxifen treatment deletes the LoxP-flanked (‘floxed’) exon 2 of Fgf10 (Urness et al., 2010) (Fig. 1A; Fig. S2), specifically in Fgf10-expressing cells themselves.

Fgf10 exon-2 wild-type, floxed and deleted alleles were respectively identified as 1.2, 1.8 and 0.45 kb products, by PCR genotyping (Roche Expand PCR) using tail biopsy-derived genomic DNA, primer pairs, Pr1, Pr2, Pr3 (Haan et al., 2013), Pr4 (5′-GAGGCAGGATAACCAGTATCTGG-3′) and Pr5 (5′-GAAATTGCAGAGATTGCAAAGGAAGC-3′) (Fig. 1A,A′C; Fig. S2), and the following cycle conditions: 1 cycle of 94°C, 2 min; 10 cycles of 94°C for 30 s, 61°C for 30 s, 68°C for 160 s, followed by 17 cycles of 94°C for 30 s, 61°C for 30 s, 68°C for 160 s plus a 40 s increment per cycle, ending with 1 cycle of 68°C for 7 min.

For sequencing, the 0.45 kb PCR fragment generated by Primers Pr4 and Pr5 was gel purified using a Thermo Fisher Scientific GeneJET PCR purification kit (K0701) and sequenced using primer Pr5 (Eurofins Genomics UK).

Both male and female mice were used, with *n* numbers provided in the main text and/or figure legends. All mice were maintained on a mixed C57BL6/129Ola genetic background and raised on Chow diet under a 12-h light/dark cycle in accordance with UK and UEA local regulations governing work with transgenic animals.

### Tamoxifen and BrdU treatments

Each pup was administered 100 µg of tamoxifen on two consecutive days by manual suckling of mouse pups with 5 µl of a stock 20 mg/ml tamoxifen solution, prepared in 10% ethanol/corn oil (Mazola) as previously described (Haan et al., 2013). Tamoxifen-pulsed pups were then returned to their mother to resume normal suckling. BrdU was administered at desired time points via intraperitoneal injections at a final concentration of 50 mg/kg body weight. Pubertal (P44) mice were administered tamoxifen on 2 consecutive days via intraperioneal injections at a concentration of 100 mg/kg body weight.

### Tissue preparation and sectioning

Animals were sacrificed by CO_2_ asphyxiation and exsanguinated transcardially with 4% paraformaldehyde solution [PFA; pH 7.4, prepared in phosphate buffered saline (PBS)]. For vibratome sectioning, brains were post-fixed in 4% PFA and dehydrated to absolute ethanol. Just before use, brains were rehydrated back to PBS, embedded in 3% agar, and sectioned on a Leica 1200 vibratome at 60 µm thickness. For cryostat sections, brains were post-fixed in 4% PFA and cryoprotected in 30% sucrose solution for 3 days at 4°C before embedding in OCT compound and sectioning at 60 µm on a Leica HM560 freezing microtome.

### Immunohistochemistry and immunofluorescence labelling

#### Antibodies

Primary antibodies used were: rabbit anti-dtTomato dsRed (1:1000; Clontech, 632496), anti-olig2 (1:500, Millipore, AB9610) and anti-cleaved caspase-3 (1:1000: Cell Signalling Technology, 9661); mouse anti-BrdU (IgG_1_; 1:200, Sigma-Aldrich, B2531), anti-GFAP (IgG_1_, 1:1000, Millipore, MAB360), anti-NeuN (IgG_1_, 1:1500, Millipore, MAB377), anti-PCNA (IgG_2a_; 1:150, Millipore, MAB424R) and anti-S100β (1:200, Abcam, Ab4066). Secondary antibodies of the relevant species and subclass, either coupled to biotin-Streptavidin Cy2 or Alexafluorochromes (-350, -488 or -568/594) were purchased from Thermo Fisher Scientific (A-11001, A-11004, A-11008 and A-11011) and Jackson ImmunoResearch (115-065-206 and 016-220-084), and used at previously described dilutions (Hajihosseini et al., 2008).

#### Immunolabelling and imaging

To simultaneously permeabilize and block non-specific binding sites, vibratome-generated sections were incubated in a solution of 20% normal goat serum (NGS) and 1% Triton X-100 (TX) for 2 h at room temperature. Sections were then incubated overnight at 4°C, with primary antibodies diluted in 0.2% NGS, 0.1% Triton in PBS. The next day, sections were washed five times (1 h per wash) in 0.2% NGS/0.1% TX and incubated overnight at 4°C with secondary antibodies diluted in a solution of 0.2% NGS/0.5% NP40. Sections were then washed six times (30 min per wash) in the same. As a prerequisite for immunodetection of BrdU, sections were pre-treated with 1 M HCl at 47°C for 30 min before the antigen blocking step (Haan et al., 2013).

For cryostat sections, non-specific binding sites were blocked by 1 h incubation in 10% NGS/0.3% TX. Primary and secondary antibodies were then applied in 10% NGS/0.3% TX solution, either overnight at 4°C or for 3 h at room temperature.

After final washes in PBS, sections were counterstained with Hoechst (0.1 mg/ml) and mounted in Vectashield (Vector Laboratories). Immunolabelled cells in both vibratome- and cryostat-generated sections were imaged as single images or within 3D reconstructions of serial *z*-stack images ranging in thickness from 0.5-2.0 µm. Images were captured using a Zeiss Axioplan 2 microscope with an Apotome attachment and analyzed using Axiovision 4.8 software.

### Cell counts and statistical analysis

Tomato+ and BrdU+ cells were counted by a single investigator under a 20× objective (×200 magnification). To avoid double counting, only whole Tomato+/Hoechst+ nuclei were counted. Cell morphology, double labelling and/or co-localization of marker antibodies in cells were verified in cut-views of 3D reconstructed images. The parenchyma was defined as the area contained within a 700 µm radius of the 3V. Random sections were double-counted by two independent workers, one of whom was blind to the experiment. The margin of error in these comparisons was below 7%.

Bregma co-ordinates of sections was determined by consultation with ‘The Mouse Brain Atlas’ (G. Paxinos & K. Franklin; 4th Edition, Elsevier), taking into consideration the shape and width of the ventricular spaces, the presence of particular hypothalamic nuclei and other brain landmarks, as well as the serial positioning of the sections with respect to other bregma-determined sections.

Significant differences were determined by subjecting the data to an unpaired two-tailed Student's *t*-test, one-way or two-way ANOVA as indicated in each figure legend, with Tukey's posthoc test. *P*<0.05 was defined as significant. All statistics were performed using Graphpad Prism software.

### Organotypic slice culture preparation and live cell imaging

DTG mouse pups were pulsed with tamoxifen at P4/P5 *in vivo* and brains were isolated at desired time points between P7 to P12, sliced and imaged for up to 96 h *ex vivo*. Briefly, brains were dissected in a medium composed of NaCl (120 mM), KCl (5 mM), CaCl_2_.2H_2_O (2 mM), MgCl_2_.6H_2_O (1 mM), NaH_2_PO_4_ (1 mM), NaHCO_3_ (1 mM), HEPES (25 mM), D-glucose (11 mM), myo-inositol (2.5 mM), 2% BME Amino acids 50× (Sigma-Aldrich, B6766), Glutamax X-100 (2 mM) and 0.1% bovine serum albumin (Fraction V, cell-culture tested), supplemented with 100 μg streptomycin and 100 units of penicillin. Brains were then embedded in UltraPure™ low melting point agarose (Thermo Fisher Scientific, 16520050) and 180 μm-thick slices were prepared by vibratome sectioning (Leica). After an hour of incubation in dissection medium at 4°C, sections were allowed to adhere to 0.4 μm pore size Millicell cell culture inserts (Merck Millipore, PICM03050) placed in 6-well plates, and bathed in 1 ml of culture medium [50% DMEM (high glucose, HEPES, lacking phenol red, Thermo Fisher Scientific, 21063029), 25% heat inactivated horse serum, 25% Hanks balanced salt solution, 100 μg of streptomycin and 100 units of penicillin]. Slices were then incubated at 37°C and 5% CO_2_ for 2 h before imaging.

Brain slices were imaged every 3 h in dissection medium at 37°C using a wide-field Zeiss Axiovert 2 microscope, returning them to conditioned culture medium in between. Serial *z*-stack images were taken and processed by de-convolution using a theoretical point spread function value on Axiovision software. We obtained 3D reconstructed images and the distance of cell migration was analyzed using ImageJ software.

## Supplementary Material

Supplementary information
