# Supplementary material for: Fibroblast growth factor 10 is a negative regulator of postnatal neurogenesis in the mouse hypothalamus
Source: Development. 2020 Jul 13;147(13):dev180950. doi: 10.1242/dev.180950 (PMC7375484; doi:10.1242/dev.180950)
Supplement: Supplementary information [file develop-147-180950-s1.pdf]

Figure S1 – Domain-restricted expression of *Fgf10* and neural stem/ progenitor cell markers in the hypothalamus of 8-day-old (P8) mice.

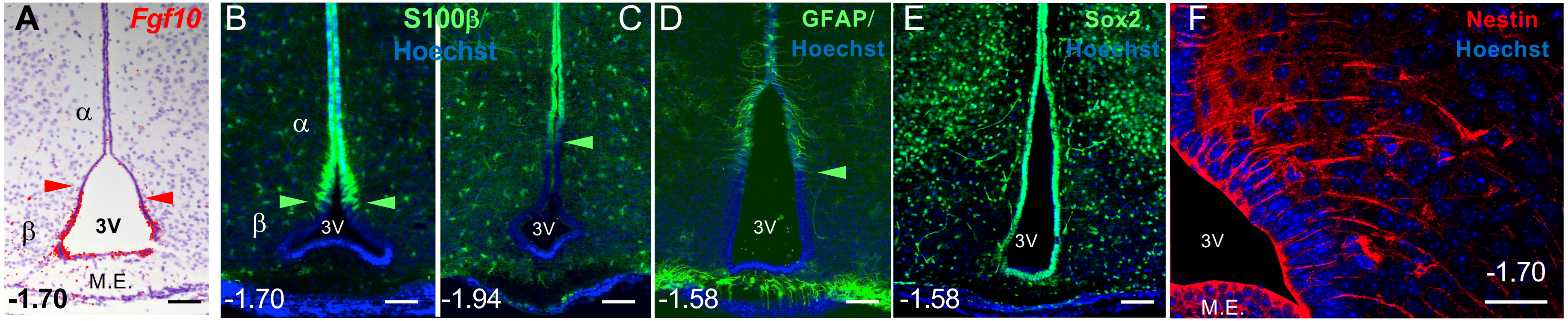

(A) Silver grains generated by isotopic in situ hybridization reaction using ^{35}S-radiolabelled Fgf10 riboprobes, false coloured to red, show strong and restricted expression pf Fgf10 to the ventral floor of 3V, corresponding to the domain of β-tanycytes. Red arrowheads, dorsal limit of Fgf10. (B-D) Immunolabelling with anti-S100β and GFAP antibodies, showing exclusion of both markers from the ventro-lateral walls of the 3V, at multiple bregma levels (see (Goodman and Hajihosseini, 2015) for S100β expression at other bregma co-ordinates). Green arrow heads point to the ventral limit of these markers. (E) Expression of SOX2 encompassing both β- and α-tanycyte domains, as well as distinct cells within the hypothalamic parenchyma. (F) High power image of right ventrolateral 3V lining, showing nestin expression by tanycytes within β2 (in M.E.) and β1 domains. Scale bars in all panel 100 μm, except F, 25 μm.

Development • Supplementary information

Figure S2 – Confirmation of Fgf10 exon 2 deletion by direct sequencing

A Pr4 →

GAAATTGCAGAGATTGCAAAGGAAGCATTTTAATTAAAACAGCATTCTCCTGTATATGC
TCAGATTCTGTACAAATGGAGGGACAACCTCTCAAAGCCTCACTCCTGGTTTGCTATACT
CCCTGGGGTTTTCAACACGTTCAACAGTTTTGGCTCAGTTTGGCTATCCCATTGTTTGTC
TTTTGCCTTGAGGATAATAGAAAATGAACTTTATCATTTGTTTTGATTTTTACCTTCTCC
CAGCTAAACATGATGACCCTTTACAGCTCATCTTTGTGATTGTGTGTTTTAG**GTGTCCTG**
GAGATAACATCAGTGGAAATCGGAGTTGTTGCCGTCAAAGCCATCAACAGCAACTATTAC
TTAGCCATGAACAAGAAGGGGAAACTCTATGGCTCAGTAAGTAGCCTGATTTTGTTTTCA
AAGCACATTGTATTGTTAGTACCAAAGATTTGGATGGTTTCCTAGAGAAGACACTGAATG
GCTTTGCTGTCATTTGATCGCTACTAATAGGATTCGCAGCATCTCAAGGGCTGGCAAACA
GCTTGTTCAACTCTATAATCTCCCGAACTGTTTGCCAATGAGTCTACTAAACAGGCCATT
ACTAACATTTGTGAATGTTCAGAACTGCAAGCCAATCAGGCTTTCACGGAAGCATCTGGA
TGAAATAGTGTAGAGACTAGAGATGACAACTTGGAGAAGAATCACTTAAAACAAAAACAT
TGGGTGGAATGGAAGATCATGCACTATGAGTATCTGCCATAGCTGAAAAGTAAAACCACA
GCTCAGATGCTGTGTACTTTGTTAAAATCATAAATAATGAAACATACCTAGGAATTTCAC
ATACCTAGGTAATAGTGTTAAATTTTAATTATGCATGAACATTATAGACATTATGTAAAA
ACAAAATACTGTCCTCACAATAGGGTCTACTTCTACCAAAAAAAAAAAACTACATGTAAT
AATAAAGGATACTCTTTTAGTAAGGTCCTCATGTGCCTTGGGTAGACTGATTAGTGTTAA
GTACTTTGAATATGTTAATATTTGAATTTGAATATTAGTGTTAAGTACTCTTCAATTTCA
AGTTCAGTTCATTTAGAAAAAGTCTTTTTGACTGAAACCTCACATAAAATAATAAGTATT
TAGAATTAAAATCACAGTCATAATTAAAACAGTATCATTCATGGTATATGATTTAAACTT
TTAGAAGAAATGTCTTAATTAAACATGTGGGTAGCTACTGTGTGCCAGATACTGGTTATC
CTGCCTC

← Pr5

B

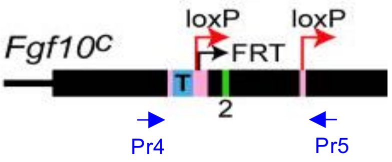

C

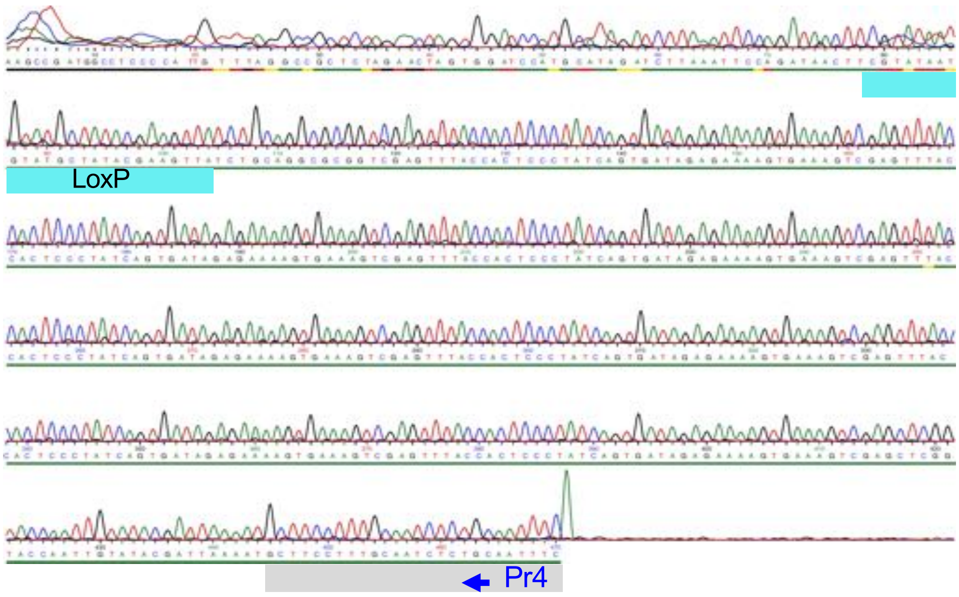

D

Multiple cloning site

LoxP

CACATGTTTAGGCCGCTCTAGAACTAGTGGATCCATGCATAGATCTTAAATTCCAGATAACT
TCGTATAATGTATGCTATACGAAGTTATCTGCAGGCGCGGTCGAGTTTACCACTCCCTATC
AGTGATAGAGAAAAGTGAAAGTCGAGTTTACCACTCCCTATCAGTGATAGAGAAAAGTGAA
AGTCGAGTTTACCACTCCCTATCAGTGATAGAGAAAAGTGAAAGTCGAGTTTACCACTCCC
TATCAGTGATAGAGAAAAGTGAAAGTCGAGTTTACCACTCCCTATCAGTGATAGAGAAAAG
TGAAAGTCGAGTTTACCACTCCCTATCAGTGATAGAGAAAAGTGAAAGTCGAGTTTACCAC
TCCCTATCAGTGATAGAGAAAAGTGAAAGTCGAGCTCGGTACCAATTGTATACGATTAAAAT
GCTTCCTTTGCAATCTCTGCAATTTC

← Pr4

(A) Mouse Fgf10 genomic sequence with nucleotides encoding exon 2 (coloured green) and its flanking introns. The position of intronic primers Pr4 and Pr5 (see also Figure 1) highlighted in blue. (B) Schematic of the targeting construct used by Urness et al. (2010) to conditionally delete Fgf10-exon2. (C,D) Sequencing trace and full nucleotide sequence of the deletion PCR product generated by Primers Pr4 and Pr5 (described in Figure 1A',C), sequenced using Primer Pr5. The position of single LoxP site that remains after deletion of exon-2 is coloured turquoise, and the position of Pr4 and residual Fgf10 nucleotides are highlighted in grey.

Figure S3 – Conditional inactivation of Fgf10 increases the number but not the relative contribution of lineage-traced cells to parenchymal ventromedial (VM) and dorsomedial (DM) nuclei.

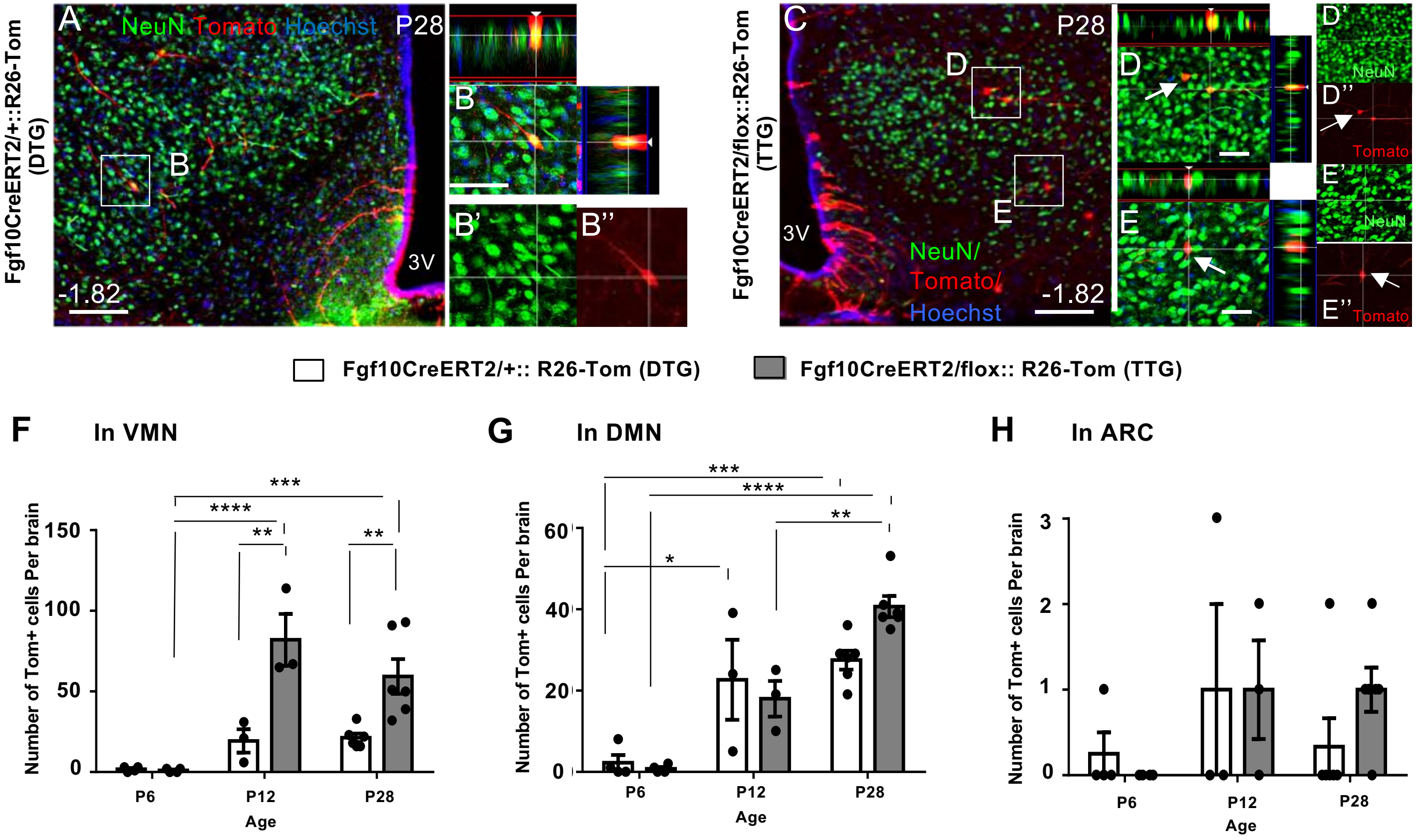

(A-E") Representative images of neurons generated by lineage-traced β-tanycytes harbouring Fgf10 (A-B'"; 'DTG') or deficient in Fgf10 (C-E"; 'TTG'). Cut views of high power images in B, D and E, showing clear co-localization of lineage-trace marker Tomato- dsred with neuronal marker, NeuN. (F-H) Numerical comparison of lineage-traced Tom+ cells in the parenchymal compartment of DTG vs TTG, showing a predominant and significant increase in contribution to VMN and DMN in both genotypes, but with relatively minimal contribution to Arc in both genotypes. For both genotypes: P6 n=4, P12 n=3, P28 n=6. Data was analysed using a two-way ANOVA followed by Tukey's test. * $p < 0.05$, ** $p < 0.01$, *** $p < 0.001$, **** $p < 0.0001$. Scale bars: A and C, 100 µm; B, D and E, 50 µm.

Figure S4 – Sagittal view: Depletion of lineage-traced Tom+ cells from the α-tanycyte domain and expression of S100β by a subset of the retained cells.

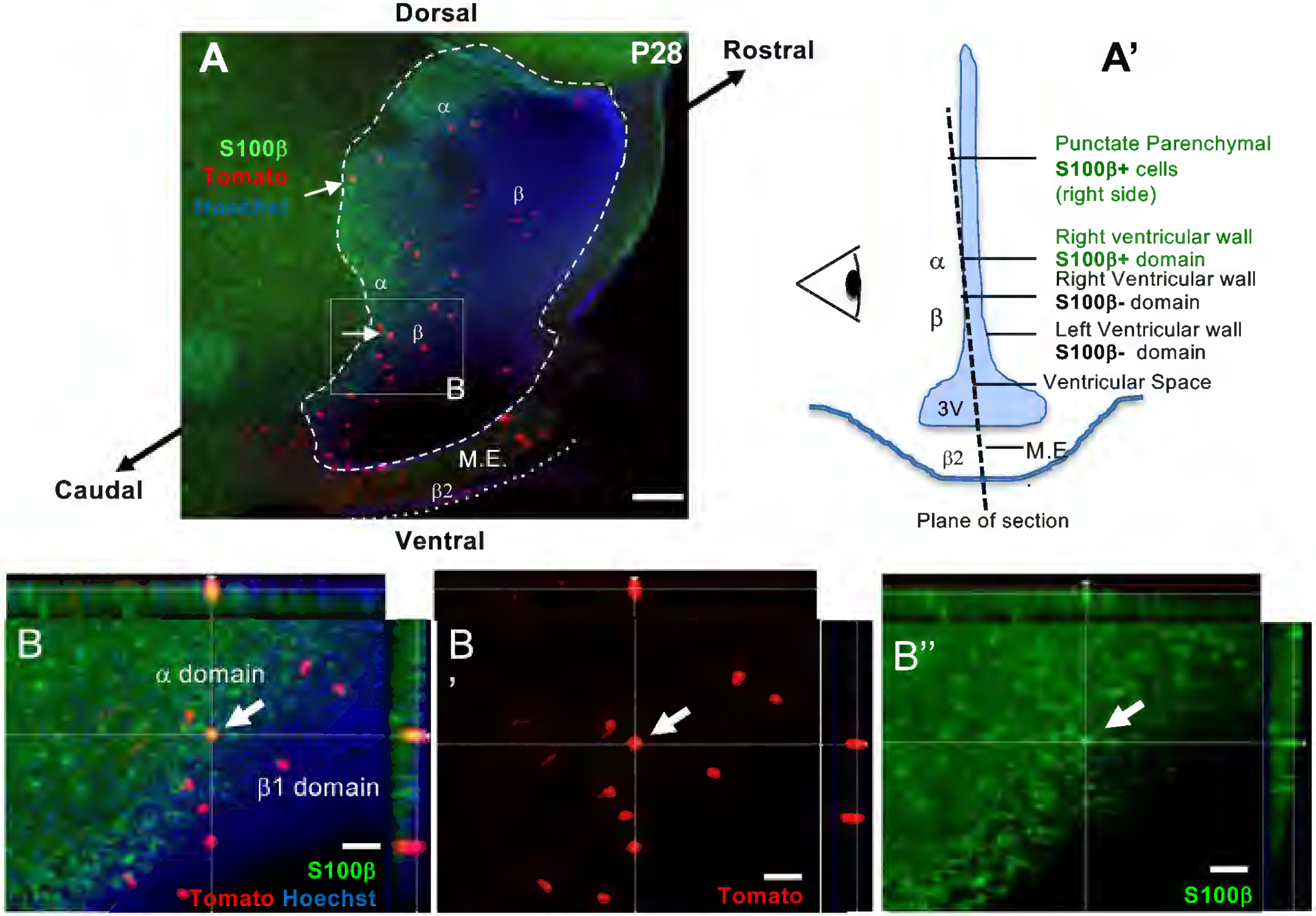

(A) A slightly oblique sagittal view of P28 hypothalamus derived from a DTG pup, tamoxifen pulsed at P4/P5, schematized in (A'). Note the paucity of lineage-traced Tom+ ependymal cells within the S100β-positive domain (green) of the ventricular wall, outlined by white dashed lines, in comparison with high retention of Tom+ cells in the S100β-negative domain, some with clear β2-tanycyte morphology in the M.E. (B-B") high power images and cut views of area 'B' showing upregulation of S100β by some Tom+ lineage-traced cells within the S-100β-positive domain of the third ventricular wall (large white arrow). Scale bars, A 100 µm, B-B", 25 µm.

Figure S5 - Paucity of cell death in early postnatal hypothalamus

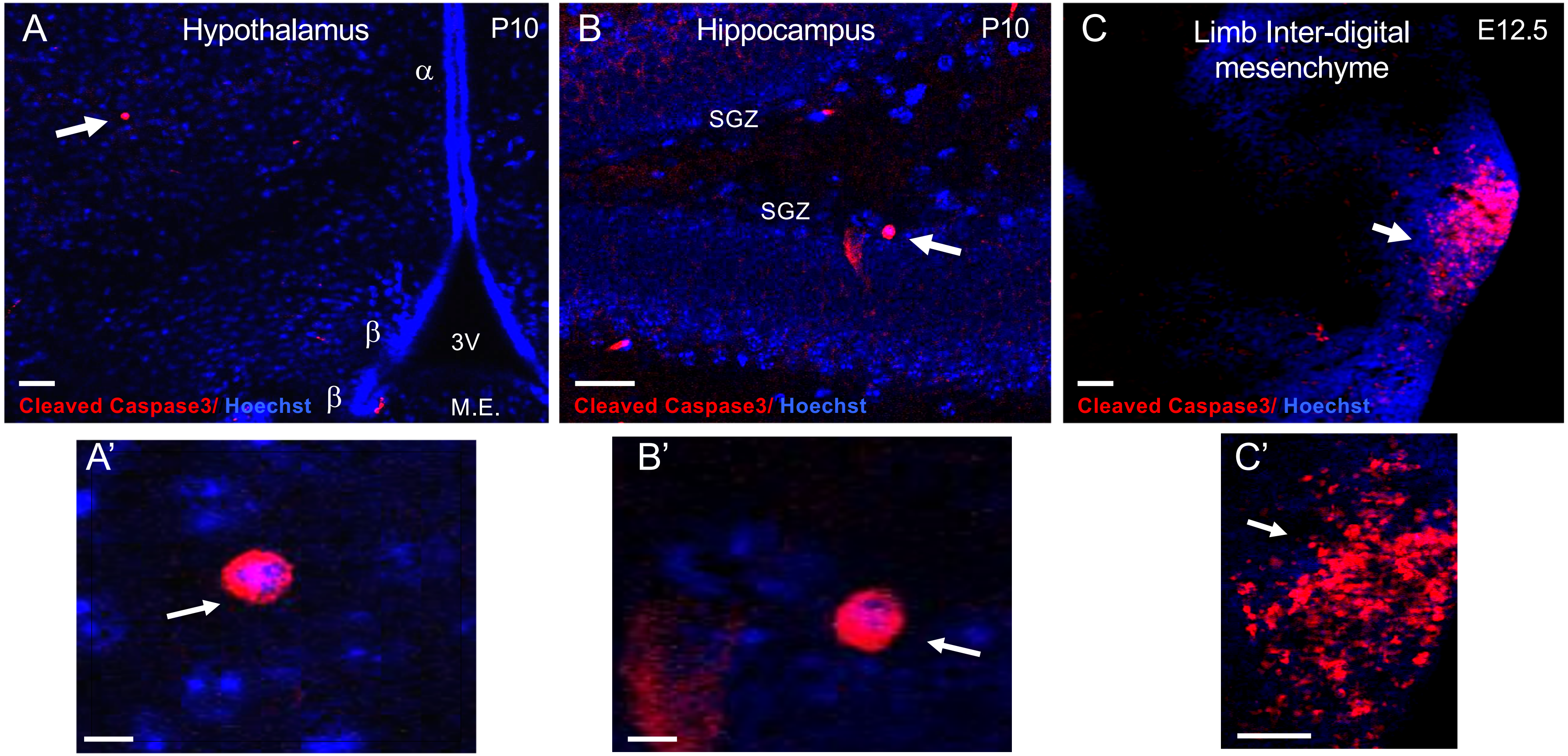

(A,A') Paucity of dying cells (white arrow) in the P10 hypothalamus in general and walls of the third ventricle in particular, as revealed by anti-cleaved caspase3 immunolabelling. (B,B') Similarly low levels of cell death in the hippocampal subgranular zone (SGZ) at P10. (C,C') Positive control tissue for anti-cleaved caspase-3 immunoreactivity. Abundance of cleaved caspase-3 positive cells in the inter-digital mesenchyme of embryonic day 12.5 (E12.5) mouse limbs, a well-established apoptotic zone during limb development (Hajihosseini et al., 2004, *Development* 131: 325-335). Scale bars, A-C,C' 50 µm, A'-B', 5 µm.

Figure S6 – β-tanycyte lineage progression and dynamics of hypothalamic neurogenesis in pubertal mice

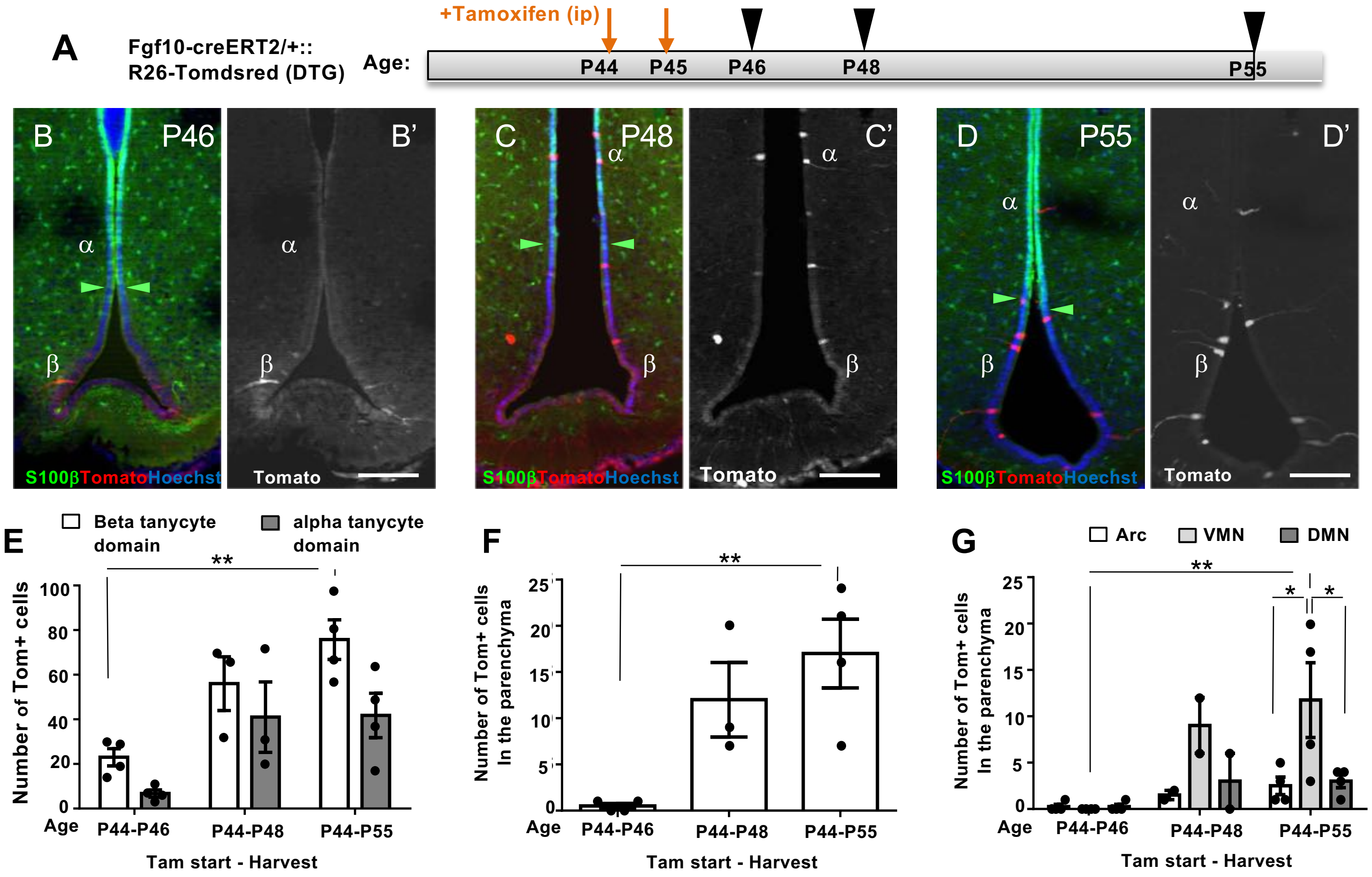

(A) Experimental Paradigm consisting of two acute tamoxifen pulses by ip at P44 and P45, and quantification of lineage traced Tom+ cells within ependymal and parenchymal compartments, either 2-4 days (P46/P48) or 9 days (P55) later. (B-D) representative images of lineage-traced tanycytes located within or outside the S100β domain at P46 (B,B'), P48 (C,C') and P55 (D,D'). Green arrow heads, ventral limit of S100β. Note the progressive appearance of Tom+ α-tanycytes within the S100β domain at P48 and their scarceness by P55. (E-F) Quantifications. (E) Note, the lagging rise of lineage-traced α-tanycytes behind β-tanycytes and the differentially higher rate of β-tanycyte retention compared to α-tanycytes. (F) Emergence of parenchymal Tom+ cells in line with peak and expansion of α-tanycytes, seen in E. (G) Predominant contribution of P44/P45 lineage traced Tom+ cells to ventromedial and dorsomedial nuclei combined, compared to the arcuate nucleus. All ages n=4 except P44-P48 n=3 (P44-P48 subdivisions n=2). (E,G) Data was analysed using a two-way ANOVA followed by Tukey's test. * $p < 0.05$, ** $p < 0.01$, and in (F) using a one-way ANOVA followed by Tukey's test ** $p < 0.01$. Scale bars, B-D' 100 µm.

Figure S7 – No discernable evidence for tomato-ds red transfer from tanycyte end feet to parenchymal cells

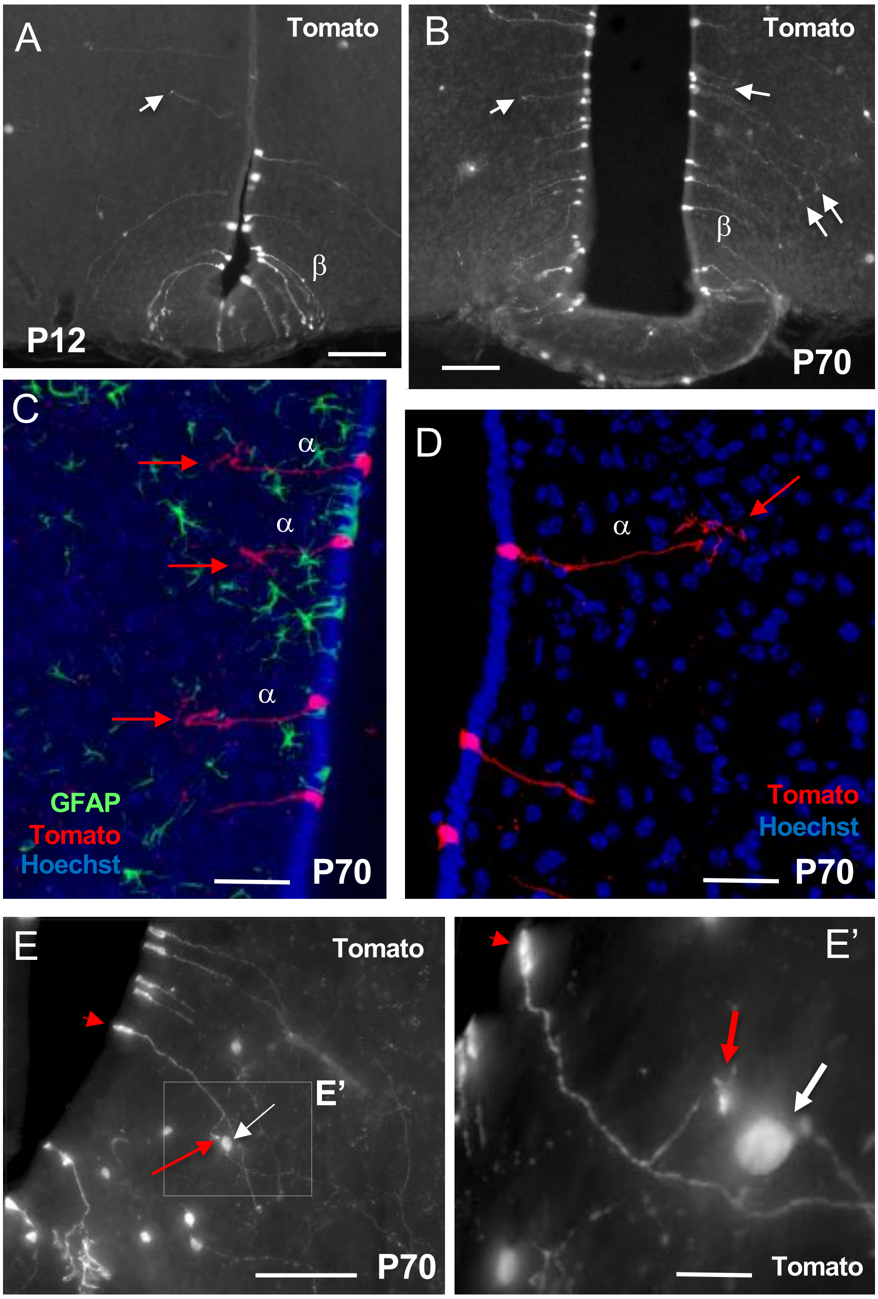

(A-E') A broad survey of beta and alpha tanycyte end feet at low and high magnification at P12, (A); and P70 (B-E'), all DTG, reveals no direct evidence for transfer of Tomato-dsred from tanycyte end feet to their immediate neighbouring cells in the parenchyma. Note the varying shapes of these end feet with some showing tortuous endings (C,D) and other with bifurcations (red arrow in E, belonging to a process of tanycyte highlighted with red arrow head). In (E and E') despite a rare close proximity of tanycyte end-foot branch to a parenchymal cell (white arrow in E'), the two are still separated by a significant gap as evident in a 3D rotation and magnified area highlighted in E'. Scale bars: A,B and E 100 µm; C, D 50 µm; and E' 10 µm.

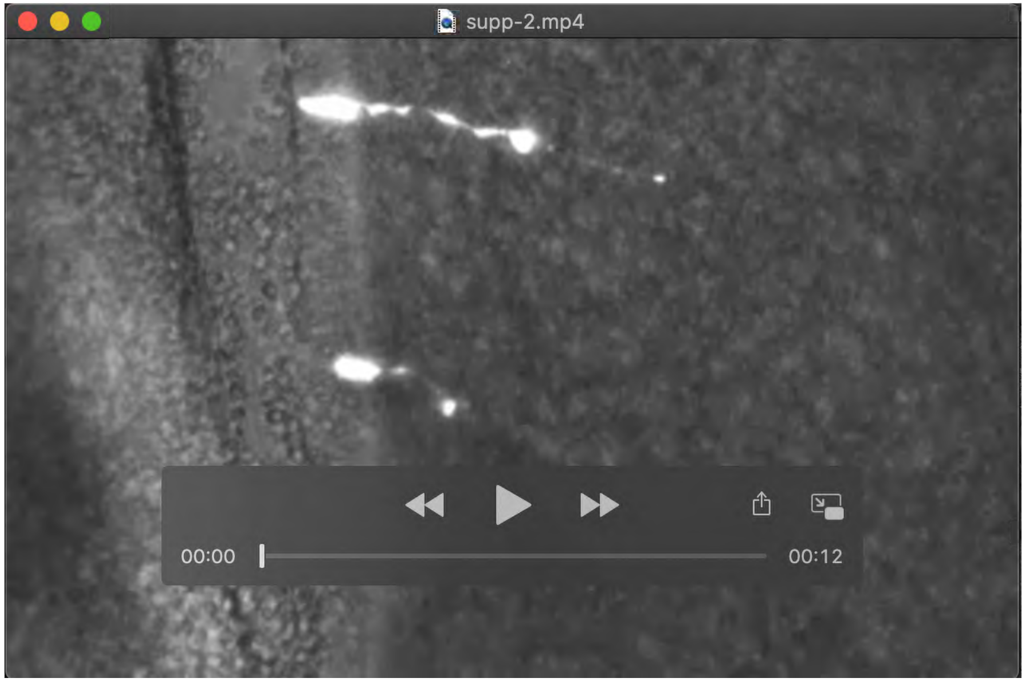

Movie 1 – Asymmetric division of lineage-traced α-tanycytes and migration of their daughters into the hypothalamic parenchyma *ex vivo*.
Movie compilation of single images obtained from the brain of a tamoxifen pulsed P4/P5 mouse, analysed at P12 *ex vivo*. Movie shows independent and asynchronous cell divisions by Tom+ lineage-traced α-tanycytes. Ventricular walls at the left of the image. Note, the lateral migration of one daughter using the radial process of the parent cell, followed by another cell division and further lateral migration of the second daughter into the hypothalamic parenchyma. Event time: 32 hours. Movie is representative of similar events observed in separate experiments and slices as well as lineage tracing studies *in vivo* (Fig. 5 E,F,H).
